# Neurocognitive effects of stress: a metaparadigm perspective

**DOI:** 10.1038/s41380-023-01986-4

**Published:** 2023-02-09

**Authors:** Eun Joo Kim, Jeansok J. Kim

**Affiliations:** 1 Department of Psychology, University of Washington, Seattle, WA 98195 USA; 2 School of Psychology, Korea University, Seoul, 02841 Republic of Korea

**Keywords:** Neuroscience, Psychology

## Abstract

Stressful experiences, both physical and psychological, that are overwhelming (i.e., inescapable and unpredictable), can measurably affect subsequent neuronal properties and cognitive functioning of the hippocampus. At the cellular level, stress has been shown to alter hippocampal synaptic plasticity, spike and local field potential activity, dendritic morphology, neurogenesis, and neurodegeneration. At the behavioral level, stress has been found to impair learning and memory for declarative (or explicit) tasks that are based on cognition, such as verbal recall memory in humans and spatial memory in rodents, while facilitating those that are based on emotion, such as differential fear conditioning in humans and contextual fear conditioning in rodents. These vertically related alterations in the hippocampus, procedurally observed after subjects have undergone stress, are generally believed to be mediated by recurrently elevated circulating hypothalamic-pituitary-adrenal (HPA) axis effector hormones, glucocorticoids, directly acting on hippocampal neurons densely populated with corticosteroid receptors. The main purposes of this review are to (i) provide a synopsis of the neurocognitive effects of stress in a historical context that led to the contemporary HPA axis dogma of basic and translational stress research, (ii) critically reappraise the necessity and sufficiency of the glucocorticoid hypothesis of stress, and (iii) suggest an alternative metaparadigm approach to monitor and manipulate the progression of stress effects at the neural coding level. Real-time analyses can reveal neural activity markers of stress in the hippocampus that can be used to extrapolate neurocognitive effects across a range of stress paradigms (i.e., resolve scaling and dichotomous memory effects issues) and understand individual differences, thereby providing a novel neurophysiological scaffold for advancing future stress research.

## Introduction

Stress is an all-encompassing term used across diverse disciplines to describe the strain on a given structure or entity. In physical sciences such as physics and engineering, stress is precisely (quantitatively) defined as the force applied per area of an inanimate object [1]. In life sciences such as biology and psychology, stress is generally (qualitatively) defined as any perturbing life situations/events—comprising both real physiological and perceived psychological stressors—that instigate adaptive bodily responses to preserve the well-being (homeostasis) of organisms [2]. While stress is an integral part of daily life, *overwhelming* adverse experiences can take their toll on physical health (e.g., cardiovascular, digestive, metabolic diseases) and mental health (e.g., anxiety, depression, post-traumatic stress disorder, schizophrenia, drug use relapse) in humans [3–6]. A common denominator of various psychopathologies linked to stress appears to be alterations in learning and memory processes, in particular the medial temporal lobe-based declarative or explicit memory that can shape and guide cognitive outlook negatively [7–10]. For instance, Holocaust survivors with posttraumatic stress disorder (PTSD) showed explicit, but not implicit, memory dysfunctions [11]. Similarly, PTSD is the primary risk factor for a higher incidence of mild cognitive impairment among the 9/11/2001 responders at the World Trade Center [12]. More recently, the stress of uncertainty and disruption to our daily lives caused by the COVID-19 pandemic has been linked to lower working and prospective (declarative) memory performances as well as higher anxiety and depression levels, which are expected to have enduring societal consequences [13, 14].

At the outset, it should be acknowledged that the majority of preclinical and clinical stress research is rooted in the use of diverse stress paradigms—for example, a single footshock stressor [15] to 210 footshock stressor [16]—characterized by adjectives such as mild, traumatic, acute, chronic, etc. Since there are no standard metrics of stress to evaluate stressors across laboratories, the resulting complex and sometimes contradictory data is difficult to make sense of. Hans Selye [17] succinctly highlighted this issue in 1973 by stating, “Everyone knows what stress is and nobody knows what it is.” Even decades later, some researchers continue to question whether stress is a useful scientific term [18]. With this caveat in mind, this review, which is admittedly narrow in scope, serves to highlight the negative effects of *inescapable and unpredictable* stress (cf. [19]; henceforth, stress) on subsequent neuronal and mnemonic functions of the hippocampus and to critically evaluate the putative role of corticosteroids synthesized and secreted by the HPA axis, which is widely acknowledged as the central stress response system [20, 21]. Whenever possible, stress techniques will be described so that the reader can assess the range of experimental outcomes. The findings and points of view discussed here could lead to new theoretical and empirical research on the neurocognitive effects of stress and offer a novel understanding of the various stress-related mental disorders that severely limit the quality of human life in today’s hectic, globalized society. Because the neurocognitive effects of stress likely vary depending on the severity of the stress and the brain region of interest, are undoubtedly mediated by multiple molecular-cellular mechanisms, and are not limited to neurons, no single review can satisfactorily integrate various findings on stress. Thus, we refer the interested reader to the following related topics and references for detailed discussion: time-dependent synaptic plasticity- and glucocorticoid-based stress models of flashbulb/traumatic memories and amnesia [6, 22], cognitive enhancing effects of mild stress [23, 24], various candidate neurochemical mediators of stress [6, 25–28], developmental stress effects [29, 30], transcriptomic-translatomic-proteomic changes to stress [31], divergent effects of stress on different brain structures [32, 33], and non-neuronal contributions in stress effects [34].

## A brief history of stress research

The detrimental effects of stress can be found anecdotally throughout human history; encountering wild animals, enemies, natural catastrophes, etc. [17, 35]. For instance, it has been suggested that King Saul in the Bible displayed “well-known signs of depression” characteristic of job insecurity stress [36], presumably resulting from the self-generated pressure of losing his kingship to a more popular David. According to the United States Library of Medicine (PubMed), the term stress first appeared in an article entitled “The Stress and Strain of Medicine” published in 1891 [37], describing the tragic suicide of Dr. Charles Edward Sheppard of the University of London, who was distressed over the death of a young patient he was administering chloroform to during an operation.

**Fig. 1:**
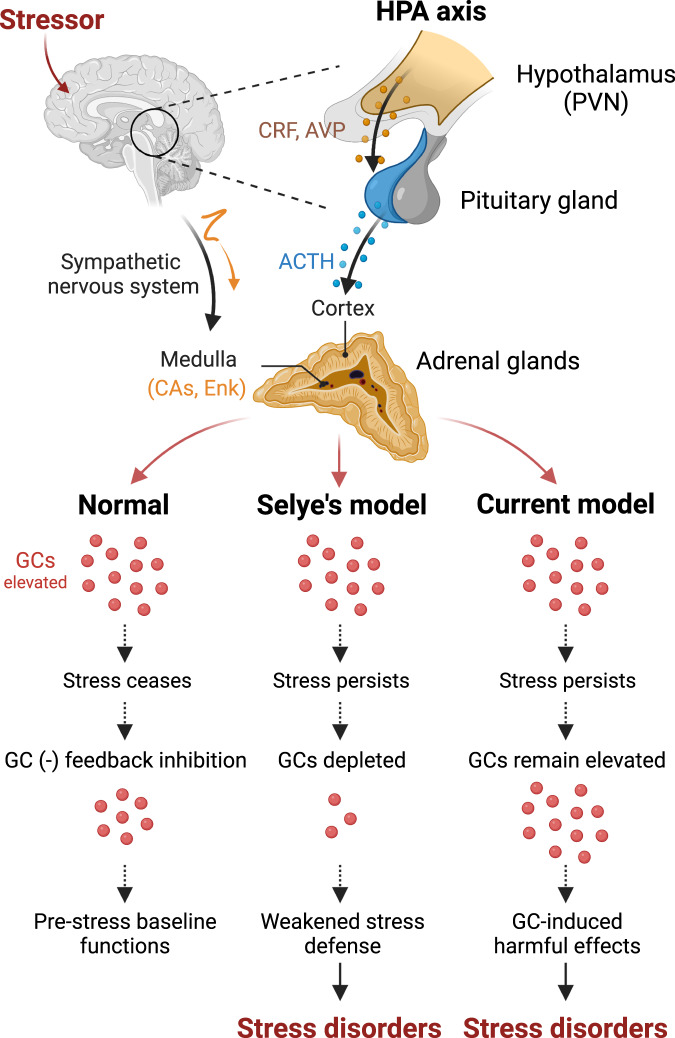
The hypothalamic-pituitary-adrenal (HPA) axis-based stress models. The HPA axis responds to physical and psychological stressors by increasing synthesis and release of various neurochemicals, namely the sympathetic nervous system-mediated rapid onset catecholamines (CAs) and enkephalins (Enk) from the adrenal medulla and relatively slower onset glucocorticoids (GCs) from the adrenal cortex. (Left) A normal, healthy response to a stressor involves elevated GCs that return to the pre-stress baseline level once the stress ceases. (Middle) Selye’s model of indirect stress effects postulated that stress-induced depletion of GCs to intense/chronic stressors makes the body susceptible to various stress-related illnesses. (Right) Current glucocorticoid model of direct stress effects contends that repetitively elevated GCs to stressors attack the body to produce stress-related illnesses. PVN paraventricular nucleus of hypothalamus, CRF corticotropin-releasing factor, AVP arginine-vasopressin, ACTH adrenocorticotropic hormone.

The modern scientific study of stress and how it affects health began decades later, in 1936, with Hans Selye’s [38] seminal paper “A syndrome produced by diverse nocuous agents.” This paper showed that rats exposed to different stressors, such as cold, surgical injury, and excessive/forced exercise, had similar physiological symptoms, such as enlarged adrenal glands, decreased thymus and spleen lymph nodes, and gastric ulceration. Based on these empirical findings, Selye proposed a biological model of stress called “general adaptation syndrome” comprising of sequential alarm reaction, adaptation and exhaustion stages and hypothesized that normal stress responses protect the organism against stressors during the alarm and adaptation stages, but depleted stress responses to severe and/or persistent stressors lead to vulnerability to stress-related illnesses (Fig. 1). However, the evidence for exhausted stress protective elements, namely the HPA axis effector corticosteroid hormones [39], to overwhelming/persistent stress was unsupported (e.g., [40]).

The general adaptation syndrome theory was then succeeded by stress models based on glucocorticoids [41–44], which still largely guide basic and clinical neuroscience research today. The glucocorticoid hypothesis of stress, while staying focused on the HPA axis effector hormones, basically reformulated Selye’s [38, 39] exhausted/depleted corticosteroid activity→stress disorder model to a heightened/protracted corticosteroid activity→stress disorder model, similar to autoantibodies attacking the body in autoimmune diseases [45]. The glucocorticoid hypothesis of stress is especially espoused in the hippocampus and its well-documented learning and memory functions (e.g., [46]). This is because hippocampal neurons are densely packed with corticosteroid-binding mineralocorticoid receptors (MRs) and glucocorticoid receptors (GRs), take part in glucocorticoid-mediated negative feedback of the HPA axis, and are sensitive to heightened corticosteroid (cortisol in human, corticosterone in rodent) actions [47]. To put it succinctly, stress is not only implicitly (neurochemically) defined as cortisol/corticosterone stress hormones, but their elevated levels are considered both necessary and sufficient to cause stress effects on the hippocampus. However, as will be discussed later, it is becoming increasingly clear that the multifaceted nature of stress cannot be simplified or emulated merely by elevated glucocorticoid levels.

## Stress effects on behavior

There is suggestive literature, predating the formal scientific studies of stress, that ‘demanding’ situations can influence behavior performances [7]. For instance, Yerkes and Dodson demonstrated in 1908 that mice given relatively challenging brightness discrimination tasks displayed a nonlinear (U-curve) learning rate as a function of shock intensity (i.e., learning was ‘favorable’ at intermediate range compared to low and high shock strengths), whereas mice given a relatively simpler brightness discrimination task displayed a linear relationship between learning rate and shock intensity [48]. Despite the fact that these early findings were inexactly represented as the Yerkes-Dodson’s inverted-U law (see [22]), they had a significant impact on the current understanding of the nonlinear relationship between stress and hippocampus function (e.g., [49, 50]). In a similar way, Pavlov [51] described *experimental neurosis* in dogs that were given increasingly difficult ‘a circle stimulus-food’ versus ‘an ellipse stimulus-no food’ discrimination conditioning. This could be seen as the first scientific example of how stress can affect learning and memory, which can lead to mental disturbances.

In the 1960s, Seligman, Overmier and Maier (see [19]) used a *triadic* experimental design with groups of dogs that underwent escapable shock, yoked inescapable shock, and control conditions. They found that the yoked inescapable shock animals (after 7-day rest) failed to show escape-avoidance learning in a novel shuttle-box. On the other hand, the escapable shock animals that received the same amount of shock but could terminate the shock showed normal escape-avoidance learning. This behavioral deficit phenomenon termed learned helplessness has been verified in numerous species, including cats, rats, fish, and humans, utilizing a variety of stressors (such as loud noise) [19]. They postulated that when a subject internalizes that its behavioral response (R) has no influence (or control) over the outcome of the aversive stimulus (S), this dissonant S-R learning results in cognitive, emotional, and motivational changes that obstruct subsequent learning of other tasks. Learned helplessness has been likened, in a contrawise manner, to Maudsley’s meta-learning, described as “the process by which learners become aware of and increasingly in control of habits of perception, inquiry, learning, and growth that they have internalized” [7].

### Stress effects on cognitive-centric tasks

Because the HPA axis responds to stressors by increasing the synthesis and release of corticosteroids that bind to corticosteroid receptor-enriched hippocampal neurons [47], substantial stress research in recent decades has focused on hippocampus-based learning and memory (Table 1). In general, it has been found that environmental stressors and exogenous cortisol/corticosterone administration can impair declarative-explicit memory in humans and spatial-relational memory in rodents [7, 52, 53]. For example, inescapable and unpredictable tone exposure (using a triadic design) impaired a human analogue escape-avoidance learning and anagram (word) solution testing in college students [54], and individuals diagnosed with PTSD showed deficits in verbal recall tasks compared to control subjects (e.g., [55, 56]). An acute hypoxic gas breathing-induced stress in healthy subjects has also been shown to impair the subsequent learning of composite memory, verbal memory, visual memory, and other declarative tasks [57]. Similarly, acute administration of cortisol in healthy subjects has been reported to selectively impair verbal (but not nonverbal) memory [58, 59], suggesting that increasing the circulating cortisol level is sufficient to mimic behavioral stress effects on declarative memory. In further accordance with the glucocorticoid hypothesis of stress, hypercortisolism associated with certain depressions and Cushing’s disease have been implicated in declarative memory deficits [60, 61].

**Table 1:** Summary of studies on stress and hippocampal-dependent and hippocampal-independent memory tasks in rats, mice, and humans.

Subject	Stress	Behavioral task	Main findings	References
Rat	Repeated restraint	Eight-arm radial maze	Impaired spatial memory	[178]
Rat	Novel environment and cat exposure	Radial arm water maze	Impaired spatial working memory	[62, 204]
Rat	Restraint + tailshocks	Morris water maze	Impaired spatial memory (hidden platform)	[66, 67]
Rat	Restraint	Contextual fear conditioning	Enhanced contextual freezing	[92]
Rat	Footshock	Morris water maze	Impaired spatial memory (hidden platform)	[71]
Rat	Restraint + tailshocks	Visual paired comparison task	Impaired long (but not short) delay recognition memory	[68]
Mouse	Repeated social confrontation	Conditional discrimination task	Impaired spatial responses	[205]
Mouse	Repeated restraint	Novel object/location task	Impaired recognition memory	[206]
Human	Combat-related PTSD (Vietnam veterans)	Wechsler memory scale, selective reminding test, and Wechsler adult Intelligence Scale	Impairments in recall, retrieval, and storage of verbal tests	[55]
Human	Combat-related PTSD (Vietnam veterans)	Auditory verbal learning test	Deficits in word-recall memory tasks.	[207]
Human	Trier Social Stress Test	A declarative memory task	Stress-induced cortisol levels correlated with declarative memory deficits	[208]
Human	Socially evaluated cold pressor	Probabilistic classification learning task	Hippocampal activity negatively correlated with performance	[209]
Human	Threat of shock	VR spatial memory task	Increased inefficient route taking	[210]
Human	Threat of shock	Word-scene association task	Impaired episodic memory retrieval.	[211]
Rat	Restraint + tailshocks	Eyeblink conditioning	Enhanced conditioned eyeblink responses.	[83, 212]
Rat	Restraint + tailshocks	Morris water maze (visible)	Enhanced habit memory (visible platform)	[67]
Rat	Chronic unpredictable stress	Instrumental task	Increased habitual strategy	[213]
Rat	Restraint	Cued fear conditioning	Enhanced cue freezing	[92]
Rat	Forced-swim Elevated platform	Delayed alternation	Enhanced working memory	[214]
Rat	Exposure to predator odor (TMT)	Dual-solution plus-maze task	Enhanced single-solution plus-maze performance	[215]
Rat	Chronic variable stress	Dual solution T-maze	Enhanced habitual learning strategy	[216]
Rat	Psychosocial	Radial arm water maze	Null effect on reference memory	[204]
Mouse	Exposure to rats	Circular hole board task	Enhanced stimulus-response strategy	[217]
Mouse	Repeated social confrontation	Conditional discrimination task	Enhanced habit-like responses	[205]
Human	Socially evaluated cold pressor test	Probabilistic classification learning task	Striatal activity positively correlated with performance	[209]
Human	Trier Social Stress Test	Spatial learning task designed by the authors	Enhanced stimulus-response learning strategy	[79]
Human	Performance pressure	Speech learning	Enhanced speech learning	[218]
Human	Mental arithmetic problems	Perceptual-motor learning task	Enhanced timings, accuracy, and variability of movement	[219]
Human	Intelligence test HAWIK-IV	Manual dexterity test (flower trail, FT)	Enhanced manual dexterity	[220]
Human	Cold pressor stress	Recall of arousing versus neutral slides	Enhanced memory for arousing but not neutral slides	[221]
Human	Grouped by Trier inventory of chronic stress	2D task	Enhanced stimulus-response strategy	[217]

Correspondingly to human studies, rats that underwent uncontrollable stress experiences exhibited memory deficits in various hippocampal-dependent tasks (for reviews, see [7, 8]). For instance, Diamond and colleagues found that 4-hr exposure to a novel environment and 30-min exposure to a cat predator selectively impaired hippocampal-dependent spatial working but not hippocampal-independent reference memory in radial arm maze tasks [62, 63]. However, a brief 2-min cat predator exposure promptly followed by radial arm watermaze training was found to enhance spatial memory, indicating a time-dependent stress effects on hippocampal functions (see [22] for a temporal dynamics model of stress and [64] for time-dependent biphasic stress effects on synaptic plasticity). Subsequent studies employing 60-min restraint+60 intermittent tailshocks and 2-hr bright light+loud noise (1-s bursts of 100 dB white noise, 30–90 s apart) also found impairments in spatial memory in a Morris water maze task [65–67] as well as object recognition memory in a visual paired comparison task ([68, 69]; see also [70]). As in the human cortisol studies mentioned above [58–61], spatial memory deficits were observed in rats subjected to acute (3.0 mg/kg, s.c.) [71] and chronic (e.g., 100 mg s.c. pellets for 1–3 months) [72] corticosteroid treatments and in trangenic mice with elevated corticosterone levels caused by the central overexpression of corticotropin-releasing factor [73]. Moreover, the stress-induced memory impairments have been found to correlate with the rise of corticosterone in the dorsal hippocampus [74]. Similar to stress effects on spatial memory in rodents, a recent study on humans found that civilians, law enforcement officers, and veterans with previous trauma exposure showed impaired spatial navigation in a virtual environment task [75].

Interestingly, the same stressors that impair hippocampal-dependent spatial memory were found to enhance the caudate-dependent response memory in rats [67, 76], mice [77], and humans [78, 79]. Since brain-memory systems are dynamic, interactive units rather than independent modules that work on their own (for a review, see [80]), it remains unclear whether the increased performance on caudate memory tasks is caused by stress directly (actively) influencing non-hippocampal-memory systems or by reducing the hippocampal memory system’s ability to compete, which indirectly (passively) enhances other brain-memory systems [67, 81].

### Stress effects on emotion-centric tasks

In contrast to its impairing effects on cognitive tasks, stress generally enhances aversive conditioning tasks in both humans and animals. In 1951, Spence and Taylor [82] reported that college students in a high-anxiety group (i.e., upper 20% anxiety scores) displayed significantly enhanced delay eyeblink conditioning (to both low- and high-intensity airpuff unconditioned stimuli) compared to those in a non-anxious group (lower 20% anxiety scores). Later, Shors and colleagues directly applied 90-min restraint+90 intermittent tailshocks to rats and found enhancements in both hippocampal-independent delay eyeblink conditioning [83] as well as hippocampal-dependent trace eyeblink conditioning [84]. It should be noted that eyeblink conditioning is thought to engage two learning processes, where rapid amygdala-based nonspecific fear response influences gradual cerebellar-based specific eyelid response acquisition (for a review, see [85]). Consistent with eyeblink conditioning, stress has been shown to enhance fear conditioning [24, 86]. For example, various stress procedures, such as 30-min and 1-hr restraint [87, 88], 15 unsignaled footshocks [89], and four days of alternating restraint+food/water restriction+predator odor+constant light+flooded cage+forced swim [90] have all been reported to increase contextual fear conditioning (cued fear conditioning was not examined in these studies). Other studies that examined contextual and tone fear conditioning in the same animals found that 1-hr restraint stress selectively enhanced contextual (but not tone) fear [91] whereas chronic restraint stress (CRS; 6-hr/day, 21 days) enhanced both contextual and tone fear [92].

Because pre-training [93] and post-training [94] lesions of the hippocampus have been shown to impair acquisition and retention, respectively, of contextual fear memory while sparing auditory fear memory, the hippocampus is thought to play an important role in processing and integrating complex context cues but not simple cues like a tone. Then, the results that stress enhances contextual fear memory vs. stress impairs spatial-relational memory seem to be at odds with each other. However, hippocampal lesioned rats can acquire contextual fear memory (especially foreground contextual fear conditioning where there is no discrete cue that competes for association with the footshock) presumably using elemental contextual cues (for a review, see [95]). Furthermore, stress-enhanced contextual fear occurs in juvenile rats (before the declarative memory system matures) and appears to be due to a nondeclarative sensitization process in adult rats (e.g., [96, 97]).

Whether stress-induced enhancement of contextual fear memory reflects hippocampal-dependent compound encoding or hippocampal-independent elemental encoding processes is yet unknown. This critical issue could be studied by subjecting hippocampal-lesioned or -inactivated animals to stress; the absence of contextual fear (or absence of enhanced contextual fear) would suggest that stress-enhanced contextual fear is likely caused by stress effects on the hippocampus, whereas the presence of enhanced contextual fear would suggest that stress-enhanced contextual fear is independent of the hippocampus. Given the evidence that the hippocampus can be functionally segmented into dorsal, intermediate, and ventral regions (for a recent review, see [98]), the effects of stress on subsequent hippocampal memory tasks can be further examined with subregion-specific hippocampal lesions or inactivation. Key stress-molecular pathways identified in hippocampal neurons (e.g., [6, 26]) can also be exploited to assess the dichotomous effects of stress on emotional vs. cognitive hippocampal memory tasks. For instance, intrahippocampal infusions of drugs that decrease/increase mitogen-activated protein kinase (MAPK) activity and subsequently decrease/increase contextual fear (similar to those shown after low and high stress) [99] would be expected to attenuate/exacerbate stress impairments of spatial-relational memory. These lines of research may advance our understanding of stress effects on fear generalization and extinction (for a recent review, see [32]), as well as fragmented trauma narratives sometimes reported in PTSD (e.g., [100]).

## Stress effects on hippocampal neurons

**Fig. 2:**
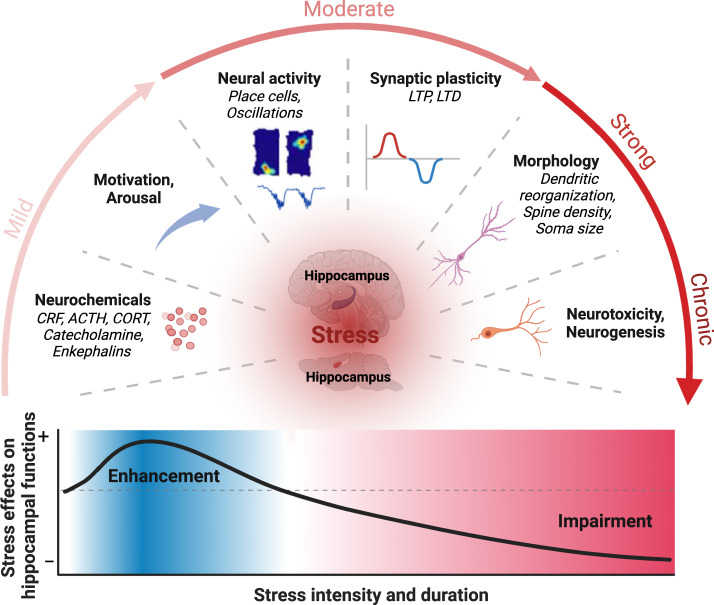
Physiological changes in the hippocampus as a function of stress magnitude. (Top) Mild stress conditions are associated with short-lived neurochemical alterations via the sympathetic nervous system, affecting motivation, arousal and alertness functions. Moderate stress conditions induce relatively longer alterations in neural activities and synaptic plasticity. Strong-chronic stress conditions can extend hippocampal alterations via impacting morphology, neurogenesis and neurotoxicity. (Bottom) These manifold physiological changes to stress can enhance or impair subsequent hippocampal mnemonic functions. The enhancing-impairing effects of stress on hippocampal functions have also been proposed by Diamond and colleagues’ “Temporal dynamics model of stress-hippocampus interactions” [22].

Acute intense and chronic stressors have been shown to cause many neurophysiological changes in the hippocampus that are postulated to subserve learning and memory mechanisms (Fig. 2).

### Synaptic plasticity

In 1987, Thompson and colleagues [101] reported that hippocampal slices prepared from rats exposed to 30-min restraint and 30 inescapable tailshocks exhibited markedly reduced long-term potentiation (LTP) in the Schaffer collateral-CA1 synapses. LTP, which refers to a sustained increase in synaptic transmission triggered by a brief high-frequency stimulation of afferent fibers, has long been considered a leading candidate synaptic model of memory in the mammalian brain [102, 103]. A triadic design study [16] then showed that rats able to escape the shock in a shuttle box (30 footshocks/day for seven days) showed reliable CA1 LTP, whereas yoked inescapable animals that received the same amount of shock showed impaired LTP, despite both groups showing comparable corticosterone elevations to shock exposure. Hence, the LTP deficit in yoked rats was essentially due to stress-associated with a lack of control over the shock, rather than nociception, fear emotion, or corticosteroid-mediated physiological effects associated with the shock [104]. Stress-induced LTP impairments have also been confirmed using in vivo CA1 recordings in awake rats placed in a novel (but not acclimatized) chamber [63, 105] and with theta-burst stimulation in anesthetized rats following 60-min restraint+60 intermittent tailshocks [106]. LTP deficits have been demonstrated in other regions of the hippocampal formation, such as the mossy fiber-CA3 synapses (in vitro) to CRS [107] and 90-min restraint+90 tailshocks [108], the perforant pathway-dentate gyrus synapses (in vivo) to CRS [109], and the dorsal CA1-subiculum synapses (in vivo) to acute 30-min restraint stress [110]. The fact that stress interferes with LTP in the hippocampus is significant because it offered a *post-hoc neurophysiological marker* to compare how different types of stress affect memory (i.e., those that impair LTP vs. those that do not).

The functional relationship between stress and LTP likely exists in nature because rats exposed to an ethologically-relevant cat predator (75-min sans physical contact) were impaired in CA1 primed burst potentiation, a low threshold form of LTP [111]. *N*-methyl-d-aspartate (NMDA) receptors and the amygdala play crucial roles because NMDA receptor antagonists and amygdala lesions/inactivation block stress impairment of LTP [65, 67, 112]. Other studies showed that stress effects on CA1 LTP last ~48/24-hr in rats/mice [113, 114] and are time-dependent and biphasic, with an initial effect that increases LTP followed by a longer-lasting effect that decreases LTP [64]; the latter parallels the 2-min cat predator-spatial memory finding mentioned above [22]. Interestingly, the same stressors (i.e., 60-min restraint+60 intermittent tailshocks [112] and brightly lit novel environment [105]) that decrease CA1 LTP have been shown to enhance CA1 long-term depression (LTD). LTD is defined by a decrease in synaptic efficacy after low-frequency stimulation of afferent fibers and is thought to work in tandem with LTP (e.g., reset LTP saturation of synapses) to process and store information (e.g., [115]). The use of different stress paradigms and afferent stimulation protocols, as well as time-dependent, biphasic effects of stress may have led to this more complicated picture of how stress affects hippocampal plasticity. Hypothetically, if LTP develops and saturates synapses in the hippocampus as a subject learns that its action and stressful situation are unrelated (i.e., learned helplessness), then subsequent LTP may be occluded while LTD is enhanced [112]. Alternatively, a metaplasticity hypothesis (i.e., the plasticity of synaptic plasticity) [116], analogous to previously mentioned meta-learning [117], has been proposed to account for the dynamic relationship between LTP and LTD with respect to stress [118, 119].

Finally, in contrast to aforementioned studies [16, 104] that found an orthogonal relationship between stress effects on LTP and corticosterone, other studies found a causal relation because stress impairment of LTP is blocked by GR antagonists [120, 121], reproduced by systemically administered corticosterone [122, 123] and observed in bath-applied corticosterone on hippocampal slices [124, 125]. Specifically, the magnitudes of LTP and corticosterone levels have been shown to have a biphasic relationship [122]. In support, the preferential activation of high-affinity MRs (reflecting mild corticosteroid levels and stress) increases LTP, while the activation of lower-affinity GRs (reflecting high corticosteroid levels and stress) decreases LTP and increases LTD [126]. The fact that NMDA receptor antagonists reduce the effects of corticosteroids [127] and prevent the effects of stress on CA1 LTP and LTD [112] suggest that Ca^2+^ influx through NMDA receptors leads to molecular-genetic cascades that change synaptic plasticity and memory functions in the hippocampus (e.g., [119]). It should be noted that corticosterone has also been shown to alter the intrinsic properties of hippocampal neurons that are not related to synaptic plasticity, i.e., corticosterone prolongs the afterhyperpolarization of CA1 pyramidal neurons via increasing the intracellular Ca^2+^ level and thereby activating the Ca^2+^-gated K + channels [128].

### Neural activity

In the rodent hippocampus are place cells that support spatial navigation by encoding memories of familiar spatial locations. Each place cell fires selectively when the animal visits a preferred location, called a *place field*, in a familiar environment [129, 130]. As an animal explores a new environment, it seems that hippocampal synaptic plasticity is involved in the formation of stable place fields because genetic and pharmacological manipulations that block LTP and spatial memory also disrupt the stability of place cells [131, 132]. Conversely, reactivation of place cell ensembles labeled with activity-dependent opsins can alter spatial navigation and memory [133]. Hence, if stress impairs LTP and spatial memory in the hippocampus, it should similarly affect the activity of place cells. Consistent with this view, rats exposed to audiogenic stress (pseudorandom 1-s bursts of 100 dB white noise) for 2 hours displayed reduced CA1 LTP, spatial memory, and place cell stability [66]. The stability of place cell activity was also found to be impacted by CRS in mice [77, 134]. Interestingly, CRS increased the local cue-dependency of place fields [77], which is in line with behavioral findings that stress enhances caudate-dependent response memory [67, 76, 77]. Lastly, a 1971 study [135] found that intraperitoneal injection of 1 mg/kg corticosterone decreases single-unit activity in the dorsal hippocampus in freely moving rats. Since then, it has been found that corticosterone increases the excitability of CA1 pyramidal cells by decreasing the amplitude of afterhyperpolarization [136, 137]. However, corticosterone (3 mg/kg) failed to change the activity of CA1 place cells in freely moving animals [138].

Stress has also been found to affect local field potentials (LFPs) in the hippocampus. LFPs are integrative excitatory and inhibitory synaptic processes [139] that span a wide range of frequencies, including theta (4–10 Hz), beta (10–30 Hz), and gamma (30–120 Hz) oscillations, as well as sharp-wave ripples (SWRs, 110–200 Hz) [140]. Rodent studies found that restraint+tailshock and CRS enhanced hippocampal theta rhythms [114] and SWRs [141] and decreased CA1 slow/fast gamma power [142]. Chronic unpredictable stress (restraint+noise+shaking+cold air stream) also heightened theta power in ventral hippocampus (vHPC) and basolateral amygdala (BLA) and increased vHPC-BLA coherence [143], whereas chronically restrained rats (2-hr/day for 10 days) exhibited decreased beta and gamma synchrony between CA1-CA3 areas but increased lateral amygdala (LA)-hippocampus synchrony with strong LA→CA1 directional activity [144]. In line with studies on animals, a recent human magnetoencephalography study found that the acute Trier Social Stress Test (public speaking stress) increased theta oscillations in the hippocampus [145]. Dynamic interactions between LFPs and spikes are thought to improve the accuracy of place coding and synchronize activities of neurons to/from multiple hippocampal efferent/afferent structures [146, 147]. However, the effect of stress on phase coupling between place cells and LFPs needs to be further investigated [148].

### Morphology and neurogenesis

In the 1990s, McEwen and colleagues [149, 150] found selective apical dendritic atrophy of CA3 pyramidal neurons (but not CA1 pyramidal neurons and dentate gyrus granule cells) in rats that either underwent CRS (6-hr/day, 21 days) or 21 days of daily corticosterone injections. A similar CA3 atrophy was observed in subordinate treeshrews subjected to chronic psychosocial stress (exposures to dominant males for 1-hr/day for 28 days) [151]. Surprisingly, the atrophied apical and basal dendrites of CA3 pyramidal neurons were found to have an increased number of dendritic spines and excresences [152], possibly reflecting compensatory mechanisms [153].

Chronic stress has also been implicated in exacerbating apoptosis and suppressing adult neurogenesis in the hippocampus [154–157]. Subordinate male (but not female) vervet monkeys were found to have fewer neurons selectively in CA3/CA2/CA1 subfields [158], gonadectomized rats that underwent restraint+water immersion for 15-min/day for 30 days showed decreased CA4/CA3 neurons [159], and rats exposed to a cat predator for 60-min/day for 2 or more weeks showed reduced CA3/CA1 neurons [160]. Studies have found that stress suppresses neurogenesis in the dentate gyrus of the hippocampus in rats that underwent CRS [161], in mice that underwent 10 social defeat episodes (in a resident-intruder test) [162], and in tree shrews and marmosets that were exposed to dominant conspecifics for 1 h [163, 164].

In accordance with animal results, magnetic brain imaging (MRI) studies have reported reduced hippocampus volume in individuals diagnosed with PTSD due to combat, childhood abuse, and workplace (i.e., police work) traumas (e.g., [165, 166] but see [167]). Moreover, the verbal declarative memory impairments were more evident in PTSD patients with smaller hippocampal volume (e.g., [165]) and decreased hippocampal activity [168]. However, because these (including poststress longitudinal) studies all measured hippocampal volume after the trauma and/or clinical diagnosis, it is not possible to dissociate traumatic events causing hippocampus reduction from having smaller hippocampus enhancing PTSD vulnerability. In fact, a monozygotic twin study [169] has suggested that reduced hippocampal volume is a predisposing factor for the development of PTSD instead of an outcome of trauma. However, Helmstetter and colleagues [170] performed a prestress-poststress longitudinal, within-subjects MRI in rats, employing the aforementioned CRS (6-hr restraint/day, 21 days) paradigm that causes dendritic atrophy, exacerbates apoptosis, and suppresses neurogenesis, and where the animals’ heads were precisely aligned and scanned under anesthesia, and found a reliable (~3%) reduction in hippocampal volume. A comparable within-subjects investigation, such as imaging the brains of enlistees before and after experiencing combat-related PTSD symptoms, may help determine whether PTSD-inducing events reduce the hippocampus in humans.

## Glucocorticoid hypothesis of stress: revisited

With Selye’s general adaptation syndrome concept empirically unsupported, the stress field underwent a renaissance with glucocorticoid-based models that continue to dominate basic and clinical research today (Fig. 1). The glucocorticoid hypothesis of stress, which is based on a single factor or class of neurochemicals, has obvious advantages in terms of parsimony, elegance, and conceptual coherence, especially when applied to hippocampal neurons densely packed with corticosteroid receptors [47, 171, 172]. As mentioned earlier, various studies have reported that glucocorticoid levels can be raised in vivo or in vitro to mimic the effects of environmental stress on the hippocampus. This includes LTP deficits [120–123, 173], dendritic atrophy [149, 150, 174], apoptosis exacerbation [156, 157], neurogenesis suppression [154, 155], and learning and memory impairments [53, 59, 62, 71, 175].

But other behavioral and neurophysiological studies disagree with this interchangeable concept of equating behavioral stress with corticosteroids (Table 2). For example, the findings of stress impairing LTP in adrenalectomized rats with dampened corticosterone levels [176] and amygdalar lesions/inactivation unaffecting stress-induced elevation of corticosterone but preventing stress impairments of LTP and spatial memory [65, 67] suggest that increased corticosterone activity is neither necessary nor sufficient to cause stress effects. The LTP was also impaired in yoked inescapable shock (but not escapable shock) animals, although the corticosterone levels were comparably elevated in the two shocked groups [16], whereas the antidepressant tianeptine blocked stress effects on LTP without affecting stress elevation of corticosterone [177]. Contrary to the glucocorticoid hypothesis, then, whether corticosteroids affect the hippocampus seems to depend on psychological conditions in which levels rise. For example, in the studies performed by McEwen and colleagues [123, 149–151, 174, 178], the animals were given corticosterone injections every day for 21 days. The animals were probably agitated because they were taken out of their homecages and had to be handled and held down for injections, both of which were out of their control and painful. Consistent with this view, male rats exposed to either sexually receptive females (appetitive condition) or a cat predator (aversive condition) showed an equivalent increase in corticosterone levels, but spatial working memory deficit was observed only under the latter condition [179]. Also, recent studies found that stress and environmental enrichment both caused corticosterone levels to rise, but they had opposite effects on the growth of new neurons in the hippocampus (see [180] for more information).

**Table 2:** Summary of studies that increased-decreased corticosterone levels (via behavioral, injection, surgical means) and resulting physiological and memory modifications in the hippocampus.

Groups	CORT	Morphology	Neurogenesis	LTP	Memory	References
Stress	↑	↓	↓	↓	↓	[16, 62, 66–68, 71, 92, 101, 112, 149, 152, 204, 222–224]
Exercise	↑	↑	↑	↑	↑	[225–231]
Sex	↑	↑	↑	↑	↑	[179, 232–238]
Feeding/Diet	↑	↑	↑	↑	↑	[239–246]
Environmental enrichment	↑	↑	↑	↑	↑	[247–250]
Adrenalectomy + Stress	↓	─	─	↓	─	[176]

Perhaps “natural” human experiments are needed to shed more light on the relationship between stress effects and corticosteroids in the real world. For example, the glucocorticoid hypothesis would predict that endurance sports events, such as the Tour de France and Vuelta a España bicycle races, involving up to six hours a day of physically and psychologically grueling cycling for 21 days, which would increase the levels of glucocorticoids to meet the metabolic demands of the body [181–183], should lead to detrimental hippocampal functioning and physiology. However, both acute and chronic exercise are usually associated with beneficial effects on the brain, cognition, and mental health [184–186]. Interestingly, after the 3-week race period, the cyclists showed decreased basal cortisol levels [187]; this finding suggests that the clinical significance of low basal cortisol condition in PTSD patients [188] may need to be reconsidered as it also occurs under non-PTSD conditions.

## Metaparadigm view of stress

The HPA axis hormones have broad functions in cell metabolism and immune responses [189, 190] and do not respond uniquely to stressful situations [8, 191]. Hence, defining stress and stress effects in terms of corticosteroids is a misnomer and overlooks the psychological complexity of stress [7, 8, 17, 18, 191]. Then, how can stress research go beyond the current HPA axis dogma?

**Fig. 3:**
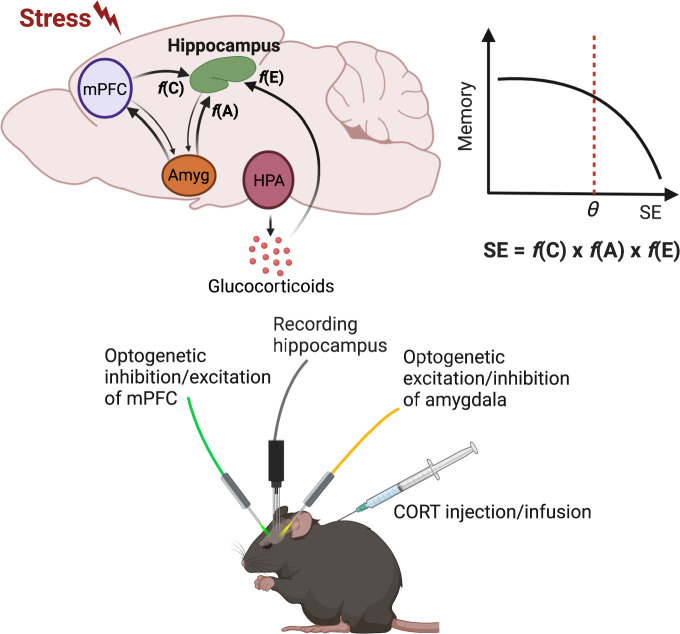
A metaparadigm view of stress-hippocampus relationship. **a** A simple neurobiological model implementing a three-component definition of stress [8]. The model hypothesizes that stress effects on the hippocampus is a product of the medial prefrontal cortex (mPFC) transmitting an inhibitory function of controllability, f(C), the amygdala (AMYG) conveying an excitatory function of aversiveness, f(A), and the corticosterone (CORT) exerting an excitatory function of excitability, f(E). **b** The hippocampal memory function declines if the stress effect (SE) exceeds a normal threshold (θ) of resilience. **c** An illustration of how a systems-level stress model can be tested in real-time by recording hippocampal neuronal activities (spikes, LFPs) in conjunctions with optogenetic-inhibition of mPFC (decrease controllability), optogenetic-stimulation of amygdala (increase aversiveness), and administering corticosterone (increase excitability) to produce/exacerbate stress effects. Conversely, optogenetic-stimulation of mPFC, optogenetic-inhibition of amygdala, and infusing corticosterone antagonists should prevent/attenuate stress effects.

Unlike in physical sciences where stress can be precisely defined, i.e., units of force per area (N/m^2^) [1], and applied across different laboratories, in life sciences there is a problem of scaling stress across laboratories because a variety of behavioral paradigms are used under the umbrella term “stress” [2]. Before neurobiology can build on a solid psychological foundation, stress needs to be clearly defined from a theoretical point of view so that it is equally applicable to human and animal studies and analyzed across behavioral paradigms, like in physical sciences. This can perhaps be achieved by advancing the learned helpless hypothesis [19], such as a three-component definition of stress that includes arousal (heightened excitability), affective (perceived aversiveness), and cognitive (lack of controllability) factors, where each is necessary but not sufficient to cause stress effects (Fig. 3; [8]). It has been further suggested that elevated levels of corticosteroids, increased activity in the amygdala, and dampened activity in the medial prefrontal cortex (mPFC) might represent the biological substrates of arousal, affective, and cognitive psychological constructs of stress, respectively [192]. Corticosteroids are linked to energy expenditure actions [43, 193], the amygdala is thought to process aversive information [65, 67, 144, 194], and the mPFC is linked to behavioral control over aversive events [195, 196], including the neuroendocrine stress response [197, 198]. Moreover, the amygdala and mPFC are ideally positioned to integrate and execute stress operations as they receive diverse sensory inputs from a number of brain regions, such as the thalamus and sensory cortex, and project to various motor output structures, such as the lateral hypothalamus for sympathetic activation, the bed nucleus of stria terminalis for stress hormones, and the periaqueductal gray for defensive behavior [199]. All of these findings support the tri-factor model of stress [7, 8]. It appears then that the multifaceted arousal-affective-cognitive notion of stress is incompatible with a single neurochemical model of stress and requires a systems-level analysis.

To date, no study has looked at how neural activities in the hippocampus change during stress in real-time in freely behaving animals and how they are reflected in subsequent learning and memory processes. Most, if not all, stress-induced physiological changes in the hippocampus we know of, such as synaptic plasticity, dendritic atrophy, apoptosis, and neurogenesis, have been observed after animals underwent stress (post-hoc), mainly in in vitro or postmortem tissue preparations. Recently, an unprecedented study by McHugh and colleagues [141] examined the dynamic effects of stress on CA1 neural activity during the first 30-min of daily immobilization stress (2-hr/day, 10 consecutive days) in mice. They found that stress decreased the overall activity of pyramidal cells but increased the synchrony between CA1 spikes and SWRs, which could be one way that synaptic saturation blocks subsequent LTP-dependent learning in the stressed hippocampus [141]. This intriguing possibility could be tested by using closed-loop, real-time interruption and prolongation of hippocampal ripples [200, 201] to reduce and increase, respectively, stress-induced alterations in hippocampal synchrony, LTP, and memory. It also remains to be determined if the onset and magnitude of this “neural signature” of stress is related to the severity of subsequent cognitive dysfunctions. Regardless, real-time monitoring of spike and LFP activities in the hippocampus, ideally simultaneously with the amygdala and mPFC, in behaving animals experiencing stress (e.g., audiogenic stress, known to alter LTP, place fields, and spatial memory; [66]), will yield new results at the neural circuit level of computations. Indeed, decoding early neural signatures of stress that reliably predict later effects on learning and memory can, in theory, serve as a new common currency across different stress paradigms and explain why stress impairs hippocampal memory in some animals but not others (individual differences) and why the same stressor impairs spatial memory but enhances contextual fear memory, when both are thought to depend on the hippocampus. Real-time analysis of how stress affects hippocampal neurons (across functionally segmented dorsal, intermediate, and ventral regions [98]) will fill crucial gaps between synaptic, cellular, and cognitive levels of analysis that are just not possible with the current post-hoc approach.

A metaparadigm approach also provides a new scaffold to experimentally manipulate the progression of stress effects, which can ultimately lead to a better understanding of and developing treatment for psychopathologies of stress. For instance, optogenetics can be applied to suppress/excite mPFC neurons and excite/suppress amygdalar neurons in conjunction with corticosteroid administration to mimic/attenuate acute stress effects on hippocampal neural activity, synaptic plasticity, and memory (Fig. 3c). Using chemogenetics [202], similar manipulations of mPFC and amygdalar neurons can be applied, except on a longer timescale, e.g., several hours daily for numerous days. This can effectively be used to induce and prevent chronic stress effects on hippocampal morphology, neurogenesis/apoptosis, and memory.

## Concluding remarks

The sine qua non of stress research has long been rooted in the HPA axis effector hormones, with the harmful effects of stress attributed initially to exhausted corticosteroid activity making the body more susceptible to illnesses [38] and then presently to persistent corticosteroid activity causing illnesses [41–44, 203]. While glucocorticoids and other supposed neurochemical [25–27] agents of stress offer a model systems approach to study stress at multiple levels of analysis, whether they accurately reflect the psychophysiological complexity of stress is questionable. For example, corticosteroids serve multifaceted functions, being involved in both aversive and appetitive situations, and thus do not respond uniquely to stress. There is also growing evidence that corticosteroids are neither necessary nor sufficient to produce stress effects in the hippocampus. Accordingly, the continued reliance on a single factor model of stress to know all about the glucocorticoids’ actions on brain and cognition will have little explanatory power; it remains unclear how biologically significant stress affects future neural and psychological processes. Perhaps Selye [17] foreseen the psychological poverty of the glucocorticoid hypothesis of stress when he concluded that “stress is not that which causes a secretion by the adrenal cortex of its hormones. ACTH, the adrenal-stimulating pituitary hormones, can discharge these hormones without producing any evidence of stress.”
